# Cytotoxicity, Bactericidal, and Antioxidant Activity of Sodium Alginate Hydrosols Treated with Direct Electric Current

**DOI:** 10.3390/ijms18030678

**Published:** 2017-03-22

**Authors:** Żaneta Król, Krzysztof Marycz, Dominika Kulig, Monika Marędziak, Andrzej Jarmoluk

**Affiliations:** 1 Department of Animal Products Technology and Quality Management, The Faculty of Food Science, Wroclaw University of Environmental and Life Sciences, Chelmonskiego 37/41, 51-630 Wroclaw, Poland; dominika.kulig@upwr.edu.pl (D.K.); andrzej.jarmoluk@upwr.edu.pl (A.J.); 2 Department of Environment Hygiene and Animal Welfare, The Faculty of Biology and Animal Science, Wrocław University of Environmental and Life Sciences, Chelmonskiego 38 C, 50-630 Wroclaw, Poland; krzysztof.marycz@upwr.edu.pl; 3 Department of Animal Physiology and Biostructure, Faculty of Veterinary Medicine, Wroclaw University of Environmental and Life Sciences, Chelmonskiego 38 C, 50-630 Wroclaw, Poland; monika.marędziak@upwr.edu.pl

**Keywords:** sodium alginate, hydrosol, direct electric current, antibacterial activity, cytotoxicity, pH, STEM, DPPH, FRAP

## Abstract

The aim of the study was to investigate the effect of using direct electric current (DC) of 0, 200, and 400 mA for five minutes on the physiochemical properties, cytotoxicity, antibacterial, and antioxidant activity of sodium alginate hydrosols with different sodium chloride concentrations. The pH, oxidation-reduction potential (ORP), electrical conductivity (EC), and available chlorine concentration (ACC) were measured. The effect of sodium alginate hydrosols treated with DC on *Staphylococcus aureus*, *Listeria monocytogenes*, *Bacillus cereus*, *Micrococcus luteus*, *Escherichia coli*, *Salmonella enteritidis*, *Yersinia enterocolitica*, *Pseudomonas fluorescence*, and RAW 264.7 and L929 cells was investigated. Subsequently, the antioxidant properties of hydrosols were evaluated by determining the scavenging ability of 1,1-diphenyl-2-picrylhydrazyl free radical (DPPH) and ferric reducing antioxidant power (FRAP). The results have shown that after applying 400 mA in hydrosol samples with 0.1% and 0.2% NaCl all tested bacteria were inactivated. The ACC concentration of C400 samples with NaCl was equal to 13.95 and 19.71 mg/L, respectively. The cytotoxicity analysis revealed that optimized electric field conditions and the addition of sodium chloride allow for the avoidance of toxicity effects on normal cells without disturbing the antibacterial effects. Due to the presence of oxidizing substances, the DPPH of variants treated with DC was lower than the DPPH of control samples.

## 1. Introduction

Pathogenic microorganisms cause infectious diseases which worldwide kill more people than any other single cause. Infections caused by pathogenic microorganisms are of great concern in many fields, for example, hospital surfaces and surgical equipment, drugs, medical devices, food packaging and storage, water purification systems, health care products, and hygienic applications [1]. Each year, an estimated 46 million people in the United States suffer from foodborne diseases which cause about 3000 deaths [2]. *Escherichia coli* (*E. coli*), *Staphylococcus aureus* (*S. aureus*), *Campylobacter jejuni* (*C. jejuni*), *Salmonella* spp., and *Listeria monocytogenes* (*L. monocytogenes*) are considered some of the most important pathogens that can cause illness and death [3]. Moreover, Yersiniosis, an infection caused by *Yersinia enterocolitica (Y. enterocolitica*), is the most frequently reported zoonotic gastrointestinal disease after campylobacteriosis and salmonellosis [4]. It has been suggested that *Bacillus cereus* (*B. cereus*) should be controlled and might be a suitable microbiological safety indicator for food products, as it causes food poisoning and some severe human infectious diseases [5]. Food manufacturers attempt to reduce or eliminate microorganisms from food products to avoid spoilage and deterioration of the quality of food products, foodborne infections, and illnesses [6]. *Pseudomonas* spp. is the most common microorganism that spoils fresh chilled meat, fish, poultry, and rabbit when the population exceeds ~10^8^ colony-forming units (CFU)/mL, it has a tendency to produce a strong spoilage smell [7,8,9]. 

Chemical solutions such as sodium hypochlorite, chlorine dioxide, hydrogen peroxide, organic acids, and ozone have been used as sanitizers in the food industry. The most commonly and widely used sanitizer is chlorinated water of 50–200 ppm [3]. Strong acid electrolyzed water (pH 2.5 ± 0.2; 20–60 mg/L available chlorine concentration (ACC)) and slightly acidic electrolyzed water (SAEW) (pH 5.0–6.5; 10–30 mg/L ACC) are novel antimicrobial agents that have been used in Japan for several years [10]. Generation of electrolyzed water involves reaction in a cell containing two electrodes, an anode and cathode, separated or not by a membrane through which a diluted salts solution passes. After applying direct electric current (DC), negatively charged ions including chloride and hydroxide, move to the anode to lose electrons and form oxygen gas, chlorine gas, hypochlorite ion, hypochlorous acid, and hydrochloric acid. Positively charged ions, including hydrogen and sodium, move to the cathode to take up electrons and form hydrogen gas and sodium hydroxide, respectively [11,12]. SAEW application minimizes the effect on human health and safety issues from Cl_2_ off-gassing, reduces corrosion of equipment, and limits phototoxic side effects while maximizing the application of hypochlorous acid species [13]. Despite great potential of electrolyzed saline solutions, there is no research on the use of hydrosols or hydrogels with antibacterial activity resulting from DC application with the exception of our studies [11,14,15].

Sodium alginate is a natural anionic polysaccharide extracted from various species of brown algae. It is composed of linear chains of 1–4 linked α-l-guluronic (G) and β-d-mannuronic (M) acid residues with free hydroxyl (OH^−^) and carboxylate (–COO^−^) groups distributed along the backbone [16]. Alginate is commonly used in food, textiles, pharmaceutical, and cosmetics industries [17]. In the food industry, alginates are used to stabilize mixtures dispersion and emulsion, as they increase viscosity and form gels, and also as packaging material. Moreover, alginate can be used in the manufacture of soft capsules and consumed as a beverage to lower blood sugar levels [18]. The use of sodium alginate or composite matrix with sodium alginate as an antibacterial material has been previously studied [19,20,21]. Because of its low toxicity, as well as its biocompatibility and biodegradability properties, sodium alginate is often applied when preparing biomedical materials, (e.g., wound dressing materials with antibacterial activity) [18,19]. In our previous paper [15], the presence of ACC in sodium alginate hydrosol treated with DC was observed. On the basis of this finding we assumed that sodium alginate treated with DC can have antibacterial activity, and this hypothesis was tested in this present work. 

Hydrosols treated with DC may have antibacterial properties and their range of application could be extended. The authors believe that sodium alginate treated with DC can be used, for example, as a new active packaging material or edible food component, which would exhibit antibacterial qualities. Moreover, it could be used as a substituent for synthetic disinfection agents. 

This study was conducted to determine the antimicrobial, cytotoxic, and antioxidant properties of sodium alginate hydrosols treated with DC. 

## 2. Results

### 2.1. Physiochemical Properties of Hydrosols 

**Figure 1:**
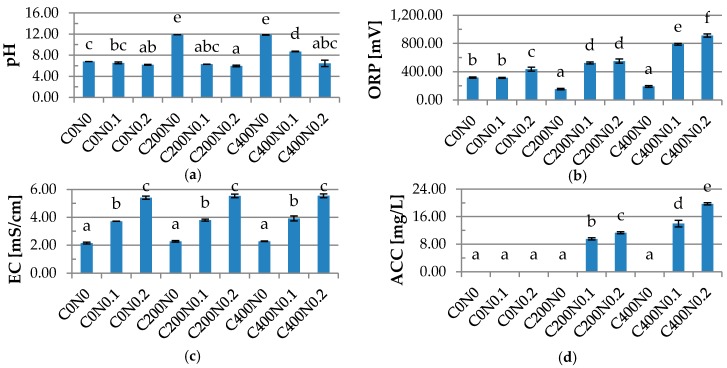
The effects of using direct electric current (DC) on the (**a**) pH; (**b**) oxidation-reduction potential (ORP); (**c**) conductivity (EC); and (**d**) available chlorine concentration (ACC) of sodium alginate hydrosols.

The characteristics of physiochemical properties of hydrosols treated by DC including pH, oxidation-reduction potential (ORP), conductivity (EC), and ACC are shown in Figure 1. After applying DC, the pH values of samples with NaCl were similar to pH measured before the treatment. The highest pH values were measured for C200N0 and C400N0 samples, 11.87 and 11.84, respectively. The lowest values of ORP were obtained for samples treated with DC without sodium chloride addition (153.15 mV and 191.1 mV, respectively), while the highest (912.55 mV) was noticed for the C400N0.2 sample. The results obtained for pH and ORP measurement can be explained by the fact that during electrolysis gel is formed on the anode. Below pKa values of the guluronic residue (pH < 3.0) sodium alginate forms gels [22]. According to Król et al. [15], gel formed on an electrode after applying 400 mA had pH of 2–3, ORP > 1100 mV, ACC of 10–90 ppm while the remaining solution had pH of 10–13 and ORP of −800 to −900 mV. After applying DC, the gel with the rest of the solution was homogenized, but the strength of the gel was large enough that it could not be completely destroyed. The higher the addition of sodium chloride, the weaker the gel becomes and the more easily it disintegrates during the homogenization. The lowest pH was measured for the C200N0.2 variant and was equal to 5.95. According to Chen et al. [22], because of the near neutral pH (5.92) of SAEW, it has fewer corrosive effects in food preparation appliances and causes less irritation to hands than acid electrolyzed water (AEW). The results obtained in our research for EC measurement confirmed that the higher concentration of electrolyte, the higher the conductivity [23]. The lowest values of EC were noticed for N0 variants (2.15–2.28 mS/cm) and the highest were measured for samples with 0.2% NaCl (5.41–5.55 mS/cm). The ACC concentration depends on the current density and sodium chloride values [24,25]. The results showed that changing the current from 200 to 400 mA caused an increase in ACC concentration from 9.53 to 13.96 mg/L for the sample with 0.1% NaCl and from 11.30 to 19.72 mg/L for the N0.2 variant. The use of DC in the hydrosol layer affects pH, ORP, and ACC parameters, while EC values depend on sodium chloride concentration. 

### 2.2. Antibacterial Properties of Hydrosols 

Table 1 shows variable counts of four G (+) and four G (−) bacteria after being treated with tested hydrosols. The results have shown that current density and addition of NaCl significantly affects the growth of bacteria. After applying DC in hydrosols with NaCl, significant reduction in all tested bacteria was observed. There were no detected bacteria for the C400N0.2 variant and for C400N0.1 except *E. coli* and *Salmonella enteritidis* (*S. enteritidis*) (about 3.90 log_10_ CFU/mL reduction). After applying 400 mA in the hydrosol layer with 0.2% NaCl, the concentration of ACC was equal to 19.72 mg/L. The increase in the addition of NaCl in hydrosols caused increased reduction of all tested microorganisms. In comparison, the application of 400 mA in the sample containing 0.1% NaCl inflicted a reduction in the *S. aureus* count of 3.91 log_10_ CFU/mL, while 0.2% NaCl resulted in a reduction of 7.06 log_10_ CFU/mL. The bactericidal effect obtained for C200N0.2 was similar to the results noticed for C400N0.1 samples, which suggests that the concentration of ACC, which strictly depends on the addition of NaCl, is the most significant antibacterial factor. The pH of C200N0.2 and C400N0.1 samples were in the range where the effective form of chlorine compounds is almost hypochlorous acid (HOCl), which has strong antimicrobial activity. Król et al. [11] showed that using weak DC directly in the hydrogel layer inhibits the growth of bacteria, and this effect is strengthened by the addition of sodium chloride to the treated medium. The inhibition zone diameter of *S. aureus* and *Y. enterocolitica* after application of 30 mA for 30 min in hydrogel with 0.5% NaCl was equal to 27.33 and 32.17 mm, respectively. Park et al. [26] studied the effect of residual chlorine and pH on the growth of *L. monocytogenes* and *E. coli* O157:H7. The results showed that antibacterial activity of the tested water increased with increasing concentrations of chlorine. Complete inactivation of *L. monocytogenes* and *E. coli* was obtained at residual chlorine levels >1.0 mg/L, pH 4.26, and ORP 895 mV and at residual chlorine levels >1.0 mg/L, pH 4.21, and ORP 915 mV, respectively. The authors assumed that the effect of chlorine on bacterial inhibition is more significant than the effect of pH. The results obtained by Vorobjeva et al. [27] showed that electrolyzed oxidizing water (EOW) with 43 mg/L of ACC caused the reduction of *B. cereus* by about 7 log CFU/mL after 1 min of treatment. Our studies revealed that a 5 min application of weak DC (200 mA) in sodium alginate hydrosol with lower ACC concentration (equal to 11.23 mg/L) also inhibited the growth of *B. cereus*. Venkitanarayanan et al. [28] tested the efficacy of EOW for inactivating *E. coli* O157:H7, *S. enteritidis*, and *L. monocytogenes*. Reduction of the population of all three pathogens by approximately 7 log CFU/mL was obtained after 5 min of treatment, while complete inactivation was noticed after 10 min of exposure. Brychcy et al. [24] investigated the effects of hydrosols incorporated with EOW on *E. coli* and *S. aureus* strains. The highest reduction of *S. aureus* and *E. coli* was observed after treatment with carrageenan (1.80 and 1.59 log reduction, respectively) and gelatin (2.10 and 1.56 log reduction, respectively) hydrosols incorporated with 0.1% of electrolyzed saline solution. Experimental gelatin and carrageenan hydrosols contained 8.20 and 2.19 mg/L of ACC, respectively. Our results indicated that using DC directly in the hydrosols layer provides better antibacterial properties. The reason for this phenomenon can be explained by the loss of ACC during preparation of hydrosols on the basis of AEW. Moreover, the temperature of hydrosols was measured before and after DC application. The results showed only a slight increase in the temperature of approximately 2 °C which did not affect the growth of bacteria.

### 2.3. Effect of Sodium Alginate Hydrosols on Cell Morphology and Intracellular Organization

**Figure 2:**
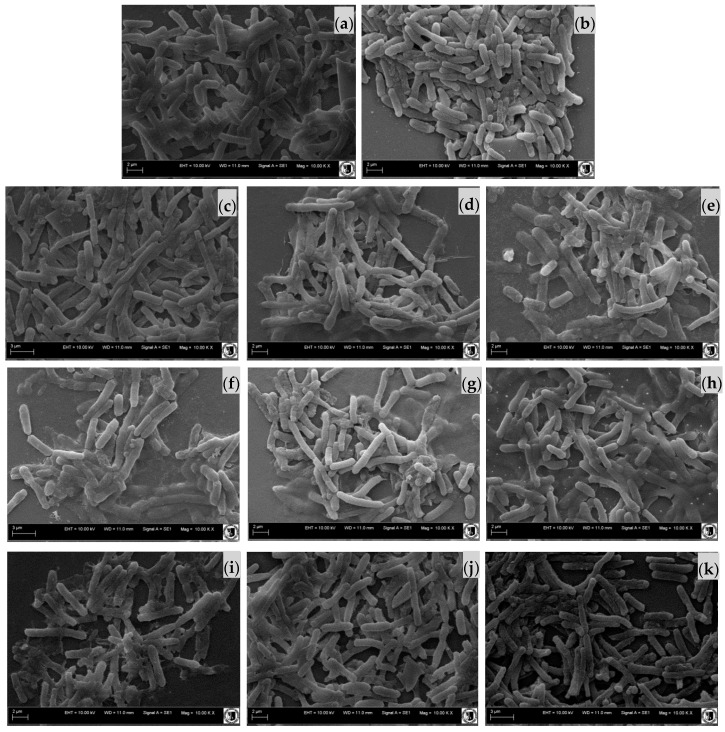
Scanning electron microscopy (SEM) of *E. coli* cells cultivated in: (**a**) Control; (**b**) SA Control (sodium alginate hydrosol with 0.9% of NaCl); (**c**) C0N0; (**d**) C0N0.1; (**e**) C0N0.2; (**f**) C200N0; (**g**) C200N0.1; (**h**) C200N0.2; (**i**) C400N0; (**j**) C400N0.1; (**k**) C400N0.2.

**Figure 3:**
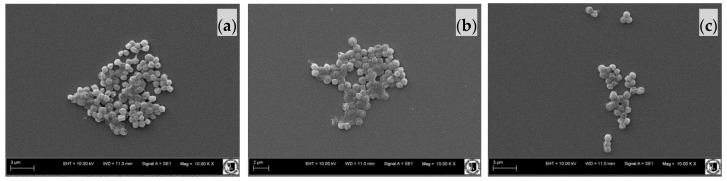
Scanning electron microscopy (SEM) of *S. aureus* cells cultivated in: (**a**) Control; (**b**) SA Control; (**c**) C400N0.2.

**Figure 4:**
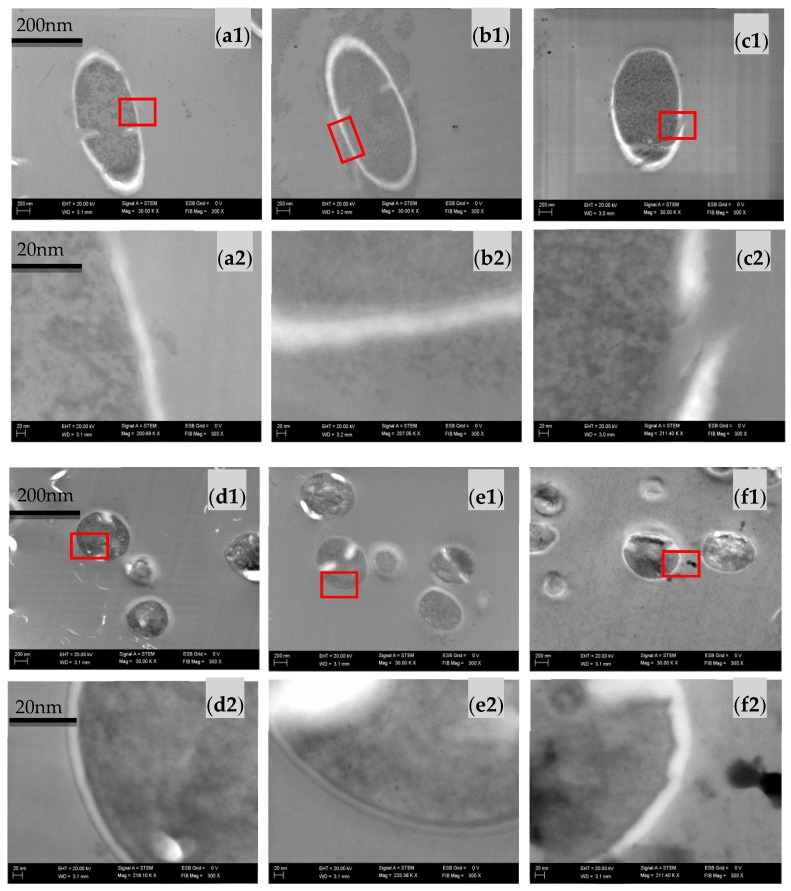
Scanning transmission electron microscopy (STEM) of *E. coli* cells cultivated in: (**a**) Control (**b**) SA Control, (**c**) C400N0.2 and *S. aureus* cells: (**d**) Control (**e**) SA Control, (**f**) C400N0.2. The number (**1**) indicates an image that shows an overview of the treated cell, while the number (**2**) indicates that an image shows an enlarged magnification of the corresponding (1) image.

The scanning electron microscopy (SEM) (Figure 2 and Figure 3) and scanning transmission electron microscopy (STEM) (Figure 4) micrographs present the surface as well as cross section of the bacterial films of *E. coli* (Figure 2 and Figure 4a1–c2) and *S. aureus* cells (Figure 3 and Figure 4d1–f2) cultivated in the investigated systems. The morphological analysis of *E. coli* (Figure 2) showed no significant difference between analyzed samples. However, the ultrastructural investigation in the C400N0.2 variant showed cell wall damage, which was visualized on STEM micrographs (Figure 4c). The morphological analysis of *S. aureus* cells of the Control as well as SA Control (sodium alginate hydrosol) revealed normal morphology (Figure 3a,b and Figure 4d,e). The significant ultrastructural differences in the surface of *S. aureus*, as well as their outer layers, were observed in C400N0.2 (Figure 4f) sample. After applying 400 mA the surface morphology changed and cell wall damage, voltage drop over the cell membrane, and irregular chromatin and cytoplasm condensation were observed. Zeng et al. [29] showed transmission electron microscopy (TEM) photos of *E. coli* and *S. aureus* treated with electrolyzed oxidizing water (EOW) for different times. After applying EO (ACC = 46.90–51.20 mg/L), there were various damages on the bacteria ultrastructure. SAEW and AEW containing ACC and high ORP effectively inactivate food-borne pathogens. However, the underlying mechanisms of inactivation remain unknown [19]. It is still not clear whether the antimicrobial potential is due to ORP, chlorine compounds, pH, or the combination of these factors [30]. The disinfection mechanism of the sodium chloride electrolyzed solution can be attributed to several factors, including the destruction of bacterial protective barriers, the increase in membrane permeability, the leakage of cellular inclusions, and the decrease in activity of some key enzymes [31]. Published studies have reported that ACC, especially HOCl, was the primary antibacterial agent in EO water. Inactivation of bacteria cells is due to chlorine-oxidizing sulfhydryl groups of certain enzymes inactivating bacteria cells [8]. It has also been proposed that chlorine causes disruption of protein synthesis and oxidative decarboxylation of amino acids to nitrites and aldehydes [10]. In general, bacteria grow in the pH range of 4–9. At pH of 5.0–6.5, the effective form of chlorine compounds in a sodium chloride electrolyzed solution is almost HOCl, which has strong antimicrobial activity. Moreover, low pH probably sensitizes the outer membranes of bacterial cells, allowing HOCl to enter the cells of bacteria more effectively [15,32]. Kim et al. [33] investigated the contribution of ORP, pH, and residual chlorine of AEW against *E. coli* 0157:H7. The authors suggest that the high ORP might be the main reason for the antibacterial properties of the tested water. This statement was confirmed by other authors [34]. High ORP in solution changes the electron flow in cells, which causes the modification of metabolic fluxes and adenosine triphosphate (ATP) production. Aerobic bacteria grow mostly at the ORP range of +200 to 800 mV, while anaerobic bacteria grow mostly at +700 to +200 mV [35]. The highest ORP was noticed for the C400N0.2 variant and was equal to 912.55 mV. However, Koseki et al. [36] noted that the ORP is not the main factor of antibacterial activity because the ozonated water with higher ORP did not show higher antibacterial activity than EO water with low ORP. The authors suggested that mainly hypochlorous acid acts on microorganisms. On the basis of our results, we also believe that hypochlorous acid is the main reason for antibacterial activity of sodium alginate hydrosols treated with DC. 

### 2.4. In Vitro Assessment of Hydrosols Treated with DC on Cytotoxicity to Mouse RAW 264.7 Macrophages and L929 Fibroblastic Cell Lines

**Figure 5:**
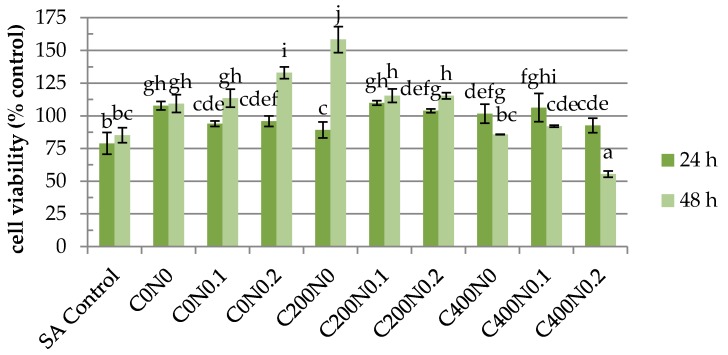
Growth curves showing proliferation rate of RAW 264.7 cells cultured on hydrosol. SA Control, sodium alginate hydrosol contained 0.9% of NaCl not treated with DC. ^a−j^ Different letters indicate significantly different groups determined by Duncan’s test (*p* < 0.05).

**Figure 6:**
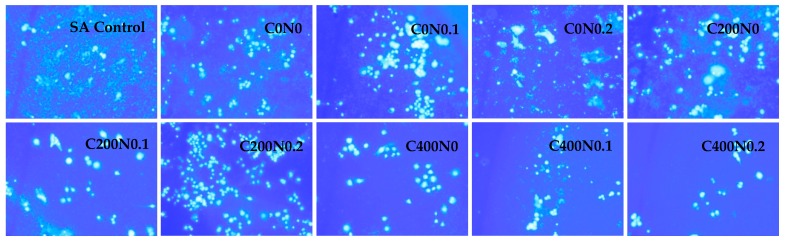
Representative microphotographs of diamidino-2-phenylindole (DAPI) staining. RAW 264.7 cells were cultured in hydrosol materials for 24 h and followed by visualization of DNA staining with DAPI using a fluorescence microscope. The data shown are representative of two individual experiments. SA Control, sodium alginate hydrosol contained 0.9% of NaCl not treated with DC.

**Figure 7:**
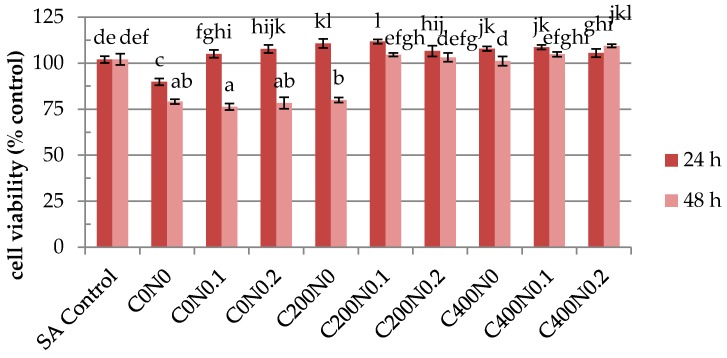
Growth curves showing proliferation rate of L929 cells cultured on hydrosol. SA Control, sodium alginate hydrosol contained 0.9% of NaCl not treated with DC. ^a−l^ Different letters indicate significantly different groups determined by Duncan’s test (*p* < 0.05).

**Figure 8:**
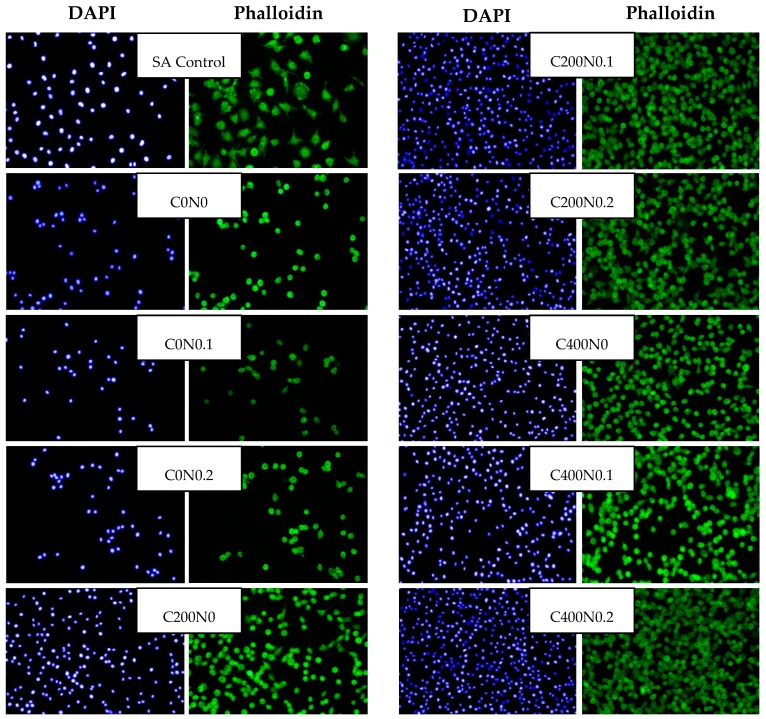
Representative microphotographs of DAPI and phalloidin fluorescent staining. L929 cells were cultured in hydrosol materials for 24. The data shown are representative of two individual experiments. SA Control, sodium alginate hydrosol contained 0.9% of NaCl not treated with DC.

The results of cytotoxicity analysis of sodium alginate hydrosols are shown in Figure 5, Figure 6, Figure 7 and Figure 8. During analysis, proper cell attachment and viability were observed. Moreover, cells grew evenly on the material surface. SA Control was included to show whether osmotic stress is one of the factors which affects the proliferation of the cells. Interestingly, the results showed that the proliferation rate of RAW 264.7 cells cultured in samples not treated with DC with 0%, 0.1%, and 0.2% NaCl were higher than for SA Control (Figure 5 and Figure 6). Different results were obtained for L929 cells after 48 h of incubation (Figure 7 and Figure 8). RAW 264.7 cells cultured for 24 h on sodium alginate hydrosols did not decrease viability below 50% of the SA Control. After 48 h, cells cultured on samples C0N0.1, C0N0.2, C200N0, C200N0.1, and C200N0.2 increased in number, whereas in the case of samples treated with 400 mA, cells slowed down their proliferation in comparison to SA Control, 24-h culture, and initial cell number (20,000 CFU). Several studies have been previously performed to evaluate the cytotoxicity of electrolyzed water solutions [37,38,39,40]. Mokudai et al. [41] suggest that intracellular reactive oxygen species (ROS) such as OH**·**, derived from HClO (Equation (1)) penetrating through the cell membrane cause the cytotoxic effects of AEW, whereas the superoxide anion radical (O_2_**·**^−^) would be derived from the mitochondria:

HOCl + O_2_**·**^−^→ OH**·** + O_2_ + Cl^−^ (1)

However, significant decrease in the cell number was observed only for the C400N0.2 sample, where the viability of cells amounted to 55.5% of the initial cell number and 65.5% of SA Control. The C400N0.2 sample had the highest ACC concentration equal to 19.72 mg/L. According to Izumi [42] and Mokudai et al. [41], the optimized electric field conditions and addition of sodium chloride allow for the avoidance of toxicity effects on normal cells while obtaining antibacterial effects. Interestingly, after applying 200 mA a greater number of cells (by ~15%) was observed in comparison to the initial number. L929 cells cultured 24 h on hydrosol increased their number above the control and SA Control (Figure 7). After 48 h, cells cultured on C200N0.1, C200N0.2, C400N0, C400N0.1, C400N0.2 samples apparently increased their proliferation in comparison to control and 24-h culture. The use of electric fields to stimulate microbial metabolism has been investigated for several decades [43]. For electrolytic stimulation 20 mA is applied to avoid bactericidal effects, which is usually observed at higher current density [44]. She at al. [45] noticed that after applying 10 mA that cell growth occurred exponentially in the first 10 h then sharply decreased, while for the controlled culture the increase in cell density continued but at a lower rate. Researchers also reported that cell growth was inhibited when 20 or 100 mA was applied. According to Nakanashi et al. [46], the exposure to a weak DC may lead to an enhanced fermentation of yeast, which is due to the oxygen generated at the anode. Comparison of the results obtained for antibacterial activity measurements with the cytotoxicity analysis revealed that the C200N0.2 variant results in a satisfactory antibacterial effect without undesirable impacts on normal cells.

The culture of L929 cells on hydrosols was characterized by heterogeneous morphology (Figure 8). Distinct cell types were present: population of fibroblast-like cells, with the predominance of bi- or multipolar cells, the most apparent of which were large flat cells of irregular shape, and small round shaped cells. The cytoskeletons of smaller fibroblast cells and large flat cells were well developed and nuclei were centrally localized, while the cytoskeletons of small cells were less developed, forming a thin rim around the oval nuclei. Cells cultured on C200N0.1, C200N0.2, C400N0, C400N0.1, and C400N0.2 samples were evenly spread on culture wells and adhered to each other. The morphology of RAW 264.7 (Figure 6) was proper in all investigated materials. The diamidino-2-phenylindole (DAPI) staining in some cases gave weak background due to the nonspecific labeling of the cytoplasm, thus it was also possible to estimate the spatial distribution of cultures. Some aggregations of nuclei were also observed in all groups. RAW 264.7 cells generally showed a round form, however, some RAW 264.7 cells in groups C200N0.1, C200N0.2, C400N0, C400N0.1, and C400N0.2 had changed to an irregular form with accelerated spreading and formed pseudopodia. This observation could explain the reduced level of cell proliferation-spreading as the pseudopodia formation could have suppressed cell proliferation.

### 2.5. Antioxidant Activity

The results of antioxidant activity analysis are shown in Table 2. Sodium alginate hydrosol (1% *v/w*) without NaCl and not treated with DC (C0N0 variant) was characterized by the highest DPPH values and was equal to 124.17 μM Trolox/mL. Results obtained by Kulig et al. [47] showed that 0.4% sodium alginate solution has DPPH values equal to ~65 μM Trolox/mL, while 0.6% has ~80 μM Trolox/mL. The results of their study alongside with our own results confirmed that sodium alginate in higher concentration has greater scavenging abilities. It was observed that the antioxidant activity of sodium alginate solution not treated with DC (C0 variants) depends on sodium chloride concentrations. The influence of the addition of sodium chloride on the antioxidant activity has been confirmed by other author [48]. It was noticed that DPPH values were higher for the C200N0 variant (97.08 μM Trolox/mL) than for the N0.1 and N0.2 variants not treated with DC (75.67 and 74.00 μM Trolox/mL, respectively). The lowest DPPH values were observed for variants with 0.2% of NaCl treated with DC (33.33–43.33 μM Trolox/mL). The above results confirmed the strong oxidizing activity of products generated during electrolyzes [49,50,51,52]. 

Ferric reducing antioxidant power (FRAP) analysis was performed to investigate the chelating ability of sodium chloride hydrosols. There were no significant differences (*p* < 0.05) between tested variants (Table 2). Similar results have been early reported by Navarro-Rico et al. [53]. The authors assumed that electrolyzed water (EW) treatment would not have any special impact on the total antioxidant activity (TAC) of the tested broccoli. Ultimately, the TAC of samples determined by the FRAP method did not show any significant change during shelf life. 

## 3. Materials and Methods 

### 3.1. Apparatus

**Figure 9:**
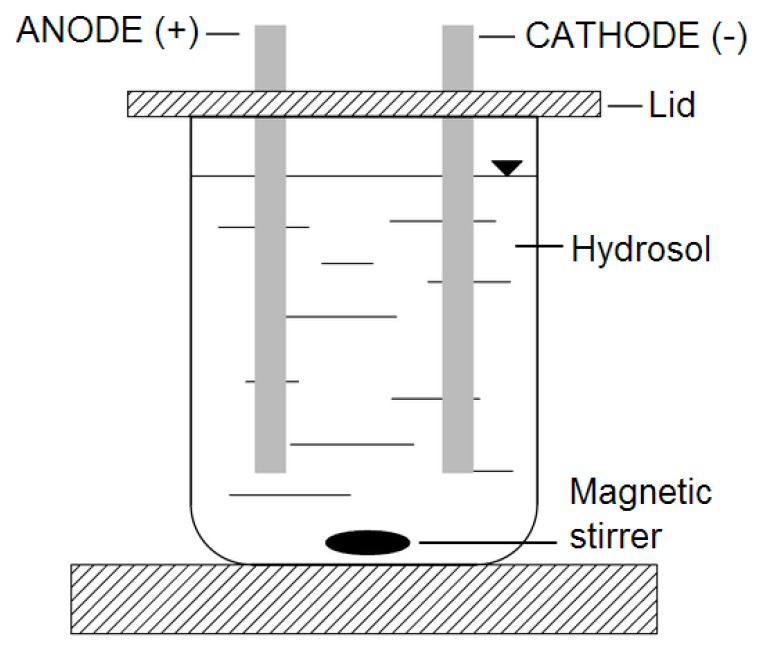
Schematic of the experimental set-up employed for the DC treatment of hydrosols.

The apparatus used to treat the samples with DC is presented in Figure 9. The electric current was provided from a DC power supply, Major Science MP-SAP (Major Science, Saratoga, NY, USA). During all the experiments, the samples were treated with DC of 0 mA, 200 mA, and 400 mA for five minutes. During DC application samples were stirred (ECM 5, CAT, Ballrechten-Dottingen, Germany) at 30 rpm. The controls were treated in exactly the same manner as the research sample, except that no electric current was applied.

### 3.2. Material

Alginate FD 125 extracted from *Laminaria digitata* (molecular weight 140 kDa, particle size max. 2% > 620 μm, M:G ratio = 1.2) was obtained from Dupont GRINSTED^®^, Grindsted, Denmark.

### 3.3. Preparation of the Experimental Material 

Sodium alginate was dissolved in sodium chloride solution in the concentrations shown in Table 3 by stirring (RW 20 digital, IKA, Staufen, Germany) at 300 rpm for thirty minutes at room temperature. After DC application, polymer solutions were homogenized by homogenizer IKA (T18 basic, Ultra Turrax, Staufen, Germany) for 15 s. The final concentration of sodium alginate hydrosols was 1% (*w/v*). 

### 3.4. Hydrosols Characterization

#### 3.4.1. Physiochemical Properties of Hydrosols

The pH, ORP and electrical conductivity (EC) of hydrosols were measured using a pH/mV/ISE Meter (Seven Multi™ model S40, Mettler Toledo, Warsaw, Poland) equipped with a pH electrode (Inlab Routine Pro, Mettler Toledo), ORP electrode (Inlab Redox Pro, Mettler Toledo), and conductivity electrode (InlabLab 731, Mettler Toledo), respectively. The available chlorine concentration (ACC, including HOCl, OCl^−^, Cl_2_, etc.) was determined by the iodometric method [54]. 

#### 3.4.2. Antibacterial Properties

The following bacterial strains were used in this study: *Staphylococcus aureus* (PCM 2602), *Escherichia coli* (PCM 2560), *Listeria monocytogenes* (PCM 2606), *Salmonella enteritidis* (m843), *Yersinia enterocolitica* (PCM 2080), *Bacillus cereus* (PCM 2003), *Pseudomonas fluorescens* (PCM 1994), and *Micrococcus luteus* (PCM 1944). These strains of microorganisms were obtained from the culture collections of the Institute of Immunology and Experimental Therapy (Polish Academy of Sciences in Wroclaw). *S. aureus*, *E. coli*, *S. enteritidis*, *Y. enterocolitica*, *B. cereus*, and *P. fluorescens* inoculum were grown in Tryptic Soy Broth (Sigma Aldrich, Poznan, Poland) at 37 °C for 18 h and at 25 °C for *M. luteus*. *L. monocytogenes* was grown in brain hart infusion broth (BHI) (Merck, Warsaw, Poland) at 30 °C. Suspensions of tested organisms were prepared in concentrations of 3.0 × 10^8^ colony-forming units (CFU)/mL by adjusting the turbidity equivalent to McFarland 1.0 standard, and the final concentrations were confirmed by agar plating. The same bacterial suspension for each bacterial species was tested throughout the investigation. To determine the antibacterial activity of the experimental hydrosols, 1 mL of the suspension was added to 9 mL of hydrosol prepared as described previously and exposed for 5 min. After 5 min treatment, bacterial suspension containing 10^7^ CFU/mL was diluted several times to obtain a dilution of 10^1^ CFU/mL and then 1 mL of each dilution was transferred to triplicate nutrient agar plates. Antibacterial activity was performed using the viable plate count method. Only plates with 30–300 colonies were counted. The control sample was sodium alginate hydrosol (SA control) with 0.9% (*w/v*) NaCl. 

#### 3.4.3. The Effect of Sodium Alginate Hydrosols on Cell Morphology and Intracellular Organization

Scanning electron microscopy (SEM) and scanning transmission electron microscopy (STEM) were used to observe the morphological changes in the tested bacteria treated with experimental hydrosols. Samples were prepared according to the method described in the work of Brychcy et al. [24]. The 18-h *E. coli* (PCM 2560) and *S. aureus* (PCM 2602) cultures were centrifuged at 5000× *g* for 10 min. The appropriate amount of sodium alginate hydrosol (25 mL of hydrosol/0.1 g of bacteria) was added to the centrifuged inoculum of the tested bacteria. After 5 min of treatment, the samples were centrifuged again (5000× *g* for 10 min). The solution was decanted and the samples were immersed in 5 mL of 2.5% glutaraldehyde. The SEM analysis was performed according to the method described by Kaliński et al. [55]. For STEM analysis, the solution was decanted and the samples were immersed in 5 mL of 2.5% glutaraldehyde in 0.1 M cacodylate buffer overnight. Afterwards, bacteria were washed three times in 0.1 M cacodylate buffer (pH = 6.8), and two times in ultrapure water, followed by resuspension of cells in a small amount of water to obtain dense bacteria suspension. Prior to vitrification, samples were aspirated to cellulose capillaries (200 μm in diameter), and the capillaries were mounted on high pressure freezing 6 mm planchettes with 20% of bovine serum albumin (BSA) for cryoprotection. Samples were vitrified by means of Leica HPM100 (Leica Microsystems, Mannheim, Germany) high pressure freezer. Frozen specimens were transferred for freeze-substitution to Leica AFS2. Freeze substitution was performed using the protocol described previously [56]. Briefly, freeze-substitution medium consisted of 95% acetone/5% water/2% osmium tetroxide/0.1% uranyl acetate. The samples were warmed from −130 °C to −90 °C for 1 h, kept at −90 °C for 10 h, and brought to −20 °C by 18 h. Afterwards, samples were placed at 4 °C for 30 min, followed by washing with pure acetone at room temperature. Next, specimens were infiltrated with Epon 812 low viscosity resin, and the capillaries collected carefully with fine tweezers and applied on fresh resin. Resin blocks were polymerized at 60 °C for 30 h. Ultrathin sections were prepared using Leica UC7 (Leica Microsystems, Mannheim, Germany) ultramicrotome and collected on TEM copper grids. Preparations were observed by means of Zeiss Auriga60 field emission-SEM (Zeiss, Jena, Germany) using STEM detector at 20 kV of acceleration voltage and bright-field mode.

#### 3.4.4. In Vitro Assessment of the Tested Hydrosols on Cytotoxicity to RAW 264.7 Macrophages and Mouse L929 Fibroblastic Cell Lines

The test was performed according to the previous protocol [57,58].

##### Cell Culture

Mouse macrophage RAW 264.7 cell lines and mouse fibroblastic L929 cell lines were purchased from American Type Culture Collection (ATCC, Manassas, VA, USA). RAW 264.7 macrophages were cultured in Dulbecco’s modified eagle medium (DMEM) 4.5 g l–d-glucose, 300 mg/L l-glutamine, and 110 mg/L sodium pyruvate with 10% fetal bovine serum, while L929 cell lines were cultured in Eagle’s Minimum Essential Medium with 10% horse serum. 

All cells were incubated at 37 °C in a humidified 5% CO_2_ atmosphere. They were observed daily under inverted light microscope (AxioObserverA1, Zeiss, Jena, Germany), images were acquired using a Cannon PowerShot, Canon, Tokio, Japan digital camera.

##### Cell Proliferation Assay

For the analysis of proliferation the hydrosols were poured into 24-well plates. The macrophage cells and fibroblastic cells were inoculated in a 1 mL volume of culture medium and seeded within the tested solutions at initial concentration 2 × 10^4^ per well. After 24 h and 48 h incubation, the effect of biomaterials was determined. Cell proliferation factor (PF) was evaluated using the resazurin based assay (TOX-8, Sigma Aldrich) according to the manufacturer’s instructions. Briefly, cell culture media were replaced with a medium containing 10% of a resazurin-based dye and incubated for two hours. Afterwards, the supernatants were collected and subjected to absorbance measurement by means of spectrophotometer (SPECTRO StarNano, BMG Labtec, Ortenberg, Germany) at 600 nm wavelength, with a distraction of 690 nm of background absorbance. To evaluate the proliferation rate and the number of live cells, a standard curve of a range of cells was calculated with absorbance directly proportional to the number of cells.

##### Cell Viability 

Cell viability was observed by light inverted and fluorescent microscope. Prior to the staining, cells were fixed with 4% ice cold paraformaldehyde for 30 min at room temperature. Then, cells were washed gently with Hank’s balanced salt solution HBSS. Washing was performed between every step of the procedure. After permeabilization of cell membranes with 0.1% Triton X-100 (15 min at room temperature), RAW 264.7 cells were incubated for 30 min in the dark with DAPI for 5 min (5 μg/mL) for the analysis of nuclei distribution. Actin filaments of L929 cells were stained using atto-488-labeled phalloidin (Sigma Aldrich) at a dilution of 1:800 with HBSS for 40 min in the dark at room temperature and the cells’ nuclei were counterstained with diamidino-2-phenylindole (DAPI; 1:1000, Sigma Aldrich). All cells were washed twice and observed under inverted phase contrast epifluorescent microscope (Zeiss, Axio Observer A.1).

### 3.5. Statistical Analysis

Each experiment was performed in triplicate. The effect of three independent categorical variables, such as the current, sodium chloride concentration, and storage time, were evaluated. Statistical analysis was performed with univariate and multivariate analysis of variance (ANOVA) using Statistica 10 (StatSoft, Cracow, Poland). The differences between the mean values were identified by the Duncan Test with a confidence level at *p* < 0.05.

#### 3.5.1. Antioxidant Activity

##### Free Radical Scavenging Activity (DPPH)

Free radical scavenging activity of the hydrosols was determined by the method of Chen et al. [59]. First, 1 mL of 0.1 mM DPPH (2,2-diphenyl-1-picrylhydrazyl)-methanol solution was incubated with 1 mL hydrosol. The reaction mixture was shaken well and incubated for 30 min at ambient temperature. The reduction of the DPPH free radicals was measured by reading the absorbance at 517 nm. The Control sample was DPPH-methanol solution. The antioxidant activity was calculated from the standard curve and expressed in μM Trolox/mL needed to neutralize 0.15 mM solution of DPPH free radicals.

##### Ferric Reducing Ion Antioxidant Power (FRAP)

The antioxidant power of hydrosols was determined by the method described by Benzie and Strain [60]. Hydrosol (100 μL) was mixed with FRAP reagent (3 mL), and absorbance was read at 593 nm. Standard calibration solutions were prepared with ferrous sulphate (0–1 mM).

## 4. Conclusions 

We investigated the antibacterial activity of sodium alginate hydrosols treated with DC against pathogenic and spoilage bacteria. Moreover, the cytotoxicity, antioxidant activity, and physiochemical properties of treated hydrosols were analyzed. The results showed that using DC of 400 mA in sodium alginate hydrosols with 0.1% and 0.2% of NaCl totally inhibited the growth of tested bacteria. The STEM micrographs show significant morphological changes in *E. coli* and *S. aureus* cells after treatment with C400N0.2 variant. Our results revealed that the higher DC levels resulted in better antibacterial activity, while worse antioxidant properties (DPPH method). The addition of NaCl was shown to enhance the antibacterial effect. After 200 mA was applied no cytotoxicity effect on RAW 263.7 cells was observed, while the inhibition of cell proliferation was noticed after treatment with 400 mA. Moreover, no cytotoxicity effect was observed (except for the C200N0 variant) after DC was applied on L929 cells after 48 h incubation. On the basis of the results obtained for antibacterial and cytotoxicity analysis, the authors believe that the C200N0.2 variant allows for a satisfactory antibacterial effect to be obtained without undesirable impacts on normal cells. 

Sodium alginate hydrosol treated by direct electric current, due to its good antibacterial properties, is the sort of material which can be used, for example, as a new active edible packaging material exhibiting antibacterial qualities. Moreover, it could be used as a substituent for synthetic disinfection agents and for medical applications (e.g., as a wound dressing with antibacterial activity).

## Figures and Tables

**Table 1:** Inactivation of different bacteria treated with hydrosols (log_10_ CFU/mL).

Variants	*S. aureus*	*L. monocytogenes*	*B. cereus*	*M. luteus*	*E. coli*	*S. enteritidis*	*Y. enterocolitica*	*P. fluorescens*
Control	7.82 ± 0.08 ^a^	7.49 ± 0.05 ^a^	7.21 ± 0.03 ^d^	7.69 ± 0.01 ^a^	7.24 ± 0.04 ^a^	7.79 ± 0.03 ^a,b^	7.46 ± 0.01 ^a^	7.77 ± 0.06 ^a^
SA Control	7.06 ± 0.04 ^b^	7.57 ± 0.03 ^a^	7.51 ± 0.01 ^a^	7.67 ± 0.01 ^a^	6.95 ± 0.00 ^b^	7.75 ± 0.01 ^a,b^	7.44 ± 0.01 ^a^	6.92 ± 0.01 ^c,d^
C0N0	6.78 ± 0.00 ^d^	7.50 ± 0.02 ^a^	7.23 ± 0.04 ^d^	7.62 ± 0.02 ^a^	6.06 ± 0.04 ^c^	7.84 ± 0.03 ^a^	7.41 ± 0.01 ^a^	6.86 ± 0.03 ^d^
C0N0.1	6.79 ± 0.01 ^d^	7.58 ± 0.07 ^a^	7.29 ±0.02 ^c^	7.65 ± 0.02 ^a^	5.95 ± 0.10 ^c^	7.69 ± 0.01 ^b^	7.41 ± 0.02 ^a^	6.96 ± 0.05 ^c^
C0N0.2	6.90 ± 0.08 ^c^	7.52 ± 0.04 ^a^	7.41 ± 0.01 ^b^	7.59 ± 0.03 ^a^	6.01 ± 0.09 ^c^	7.69 ± 0.11 ^b^	7.44 ± 0.01 ^a^	7.03 ± 0.05 ^b^
C200N0	6.63 ± 0.06 ^f^	6.75 ± 0.05 ^b^	6.21 ± 0.06 ^e^	6.72 ± 0.02 ^b^	5.37 ± 0.02 ^e^	6.63 ± 0.00 ^d^	7.33 ± 0.01 ^b^	6.95 ± 0.03 ^c^
C200N0.1	4.59 ± 0.00 ^g^	1.80 ± 0.28 ^c^	4.21 ± 0.01 ^g^	2.33 ± 0.17 ^d^	5.35 ± 0.10 ^e^	4.59 ± 0.09 ^e^	1.65 ± 0.02 ^d^	ND ^f^
C200N0.2	ND ^h^	ND ^d^	ND ^h^	ND ^e^	3.38 ± 0.01 ^f^	3.49 ± 0.01 ^g^	1.01 ± 0.05 ^e^	ND ^f^
C400N0	6.68 ± 0.05 ^e^	6.59 ± 0.21 ^b^	5.26 ± 0.02 ^f^	5.70 ± 0.12 ^c^	5.74 ± 0.03 ^d^	6.54 ± 0.06 ^c^	7.22 ± 0.05 ^c^	6.63 ± 0.01 ^e^
C400N0.1	ND ^h^	ND ^d^	ND ^h^	ND ^e^	3.35 ± 0.17 ^f^	3.88 ± 0.03 ^f^	ND ^f^	ND ^f^
C400N0.2	ND ^h^	ND ^d^	ND ^h^	ND ^e^	ND ^g^	ND ^h^	ND ^f^	ND ^f^

^a–h^ Different letters indicate significantly different groups determined by Duncan’s test (*p* < 0.05); SA Control, sodium alginate hydrosol contained 0.9% of NaCl not treated with direct electric current (DC); ND, no detectable survivors by a direct plating procedure. CFU: colony forming units.

**Table 2:** Antioxidant activity of hydrosols.

Variants	DPPH (μM Trolox/mL)	FRAP (μM Fe(II)/mL)
C0N0	124.17 ± 3.04 ^e^	1.00 ± 0.03 ^a^
C0N0.1	75.67 ± 6.81 ^c^	0.99 ± 0.04 ^a^
C0N0.2	74.00 ± 5.35 ^b,c^	1.01 ± 0.04 ^a^
C200N0	97.08 ± 15.36 ^d^	0.98 ± 0.01 ^a^
C200N0.1	62.25 ± 8.82 ^b^	0.97 ± 0.03 ^a^
C200N0.2	43.33 ± 3.19 ^a^	1.00 ± 0.03 ^a^
C400N0	75.42 ± 11.66 ^c^	0.99 ± 0.02 ^a^
C400N0.1	36.67 ± 6.16 ^a^	1.00 ± 0.01 ^a^
C400N0.2	33.33 ± 2.89 ^a^	1.00 ± 0.01 ^a^

^a–e^ Different letters indicate significantly different groups determined by Duncan’s test (*p* < 0.05).

**Table 3:** The composition of hydrosols.

Run Code Letters	Current (C) (mA)	NaCl (N) (%)
C0N0	0	0.0
C0N0.1		0.1
C0N0.2		0.2
C200N0	200	0.0
C200N0.1		0.1
C200N0.2		0.2
C400N0	400	0.0
C400N0.1		0.1
C400N0.2		0.2
